# Multicellular Cell Seeding on a Chip: New Design and Optimization towards Commercialization

**DOI:** 10.3390/bios12080587

**Published:** 2022-08-01

**Authors:** Trieu Nguyen, Linh Ho, Sakib M. Moinuddin, Tanoy Sarkar, Dipongkor Saha, Fakhrul Ahsan

**Affiliations:** 1 Department of Pharmaceutical and Biomedical Sciences, College of Pharmacy, California Northstate University, Elk Grove, CA 95757, USA; trieu.nguyen@cnsu.edu (T.N.); linh.ho@cnsu.edu (L.H.); sakib.moinuddin@cnsu.edu (S.M.M.); tanoy.sarkar@cnsu.edu (T.S.); 2 East Bay Institute for Research & Education (EBIRE), Mather, CA 95655, USA; dipongkor.saha@cnsu.edu; 3 MedLuidics, Elk Grove, CA 95757, USA

**Keywords:** microfluidics, pulmonary arterial hypertension (PAH), cell seeding, micro-milling, clean room, endothelial cells, smooth muscle cells

## Abstract

This paper shows both experimental and in-depth theoretical studies (including simulations and analytical solutions) on a microfluidic platform to optimize its design and use for 3D multicellular co-culture applications, e.g., creating a tissue-on-chip model for investigating diseases such as pulmonary arterial hypertension (PAH). A tissue microfluidic chip usually has more than two channels to seed cells and supply media. These channels are often separated by barriers made of micro-posts. The optimization for the structures of these micro-posts and their spacing distances is not considered previously, especially for the aspects of rapid and cost-efficient fabrication toward scaling up and commercialization. Our experimental and theoretical (COMSOL simulations and analytical solutions) results showed the followings: (i) The cell seeding was performed successfully for this platform when the pressure drops across the two posts were significantly larger than those across the channel width. The circular posts can be used in the position of hexagonal or other shapes. (ii) In this work, circular posts are fabricated and used for the first time. They offer an excellent barrier effect, i.e., prevent the liquid and gel from migrating from one channel to another. (iii) As for rapid and cost-efficient production, our computer-aided manufacturing (CAM) simulation confirms that circular-post fabrication is much easier and more rapid than hexagonal posts when utilizing micro-machining techniques, e.g., micro-milling for creating the master mold, i.e., the shim for polymer injection molding. The findings open up a possibility for rapid, cost-efficient, large-scale fabrication of the tissue chips using micro-milling instead of expensive clean-room (soft) lithography techniques, hence enhancing the production of biochips via thermoplastic polymer injection molding and realizing commercialization.

## 1. Introduction 

**Figure 1:**
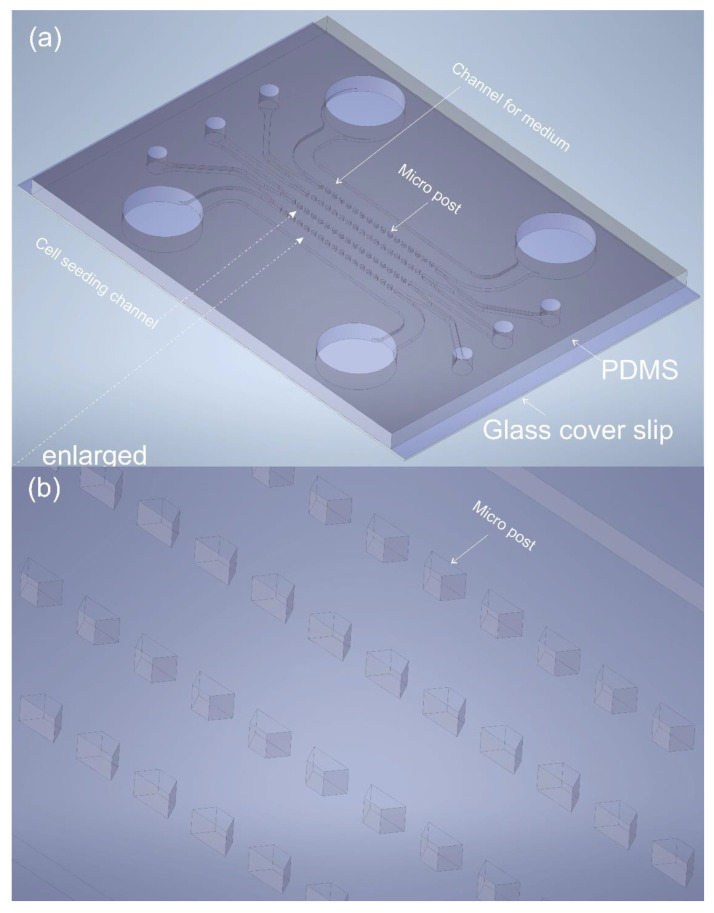
(**a**) 3D illustration of a typical tissue chip made of PDMS (bonded to a glass coverslip). Each main microchannel used for cell culture comprises two parallel rows of micro trapezoidal posts. Trapezoidal posts have been used in many publications including ours [4] and others [8,14]. (**b**) An enlargement view at the micro-post position in the chip shown in Figure 1a.

3D cell culturing in a microfluidic device has many advantages compared with conventional 2D culture methods on a flask or a Petri disk. Among the most beneficial aspects are the followings: (i) A cost reduction in the chemicals and cells used in the experiments since down-scaling into microscale. The use of chemical, biomaterial volume (e.g., media, cells) is in the order of 1 μL (or smaller) compared with mL in the conventional culture methods. (ii) The 3D structures of a microchannel offer a micro-environmental model closer to an in-vivo. (iii) Multicellular cultures and cell–cell communication are made possible. Over the past 15 years, many researchers have performed experiments on microfluidic cell seeding and culture for one cell type [1,2,3] and multiple cell types [4,5,6,7,8,9] to investigate cell–microenvironment interaction, cell–cell communication, cell migration, etc. The microfluidic chip designs in these studies have been inspiring for the current works performed in our group, such as the studies of pulmonary arterial hypertension (PAH)-on-a-chip [4]. The typical material for making the biochip in these works over many years is Polydimethylsiloxane (PDMS), and the typical design for the microfluidic components is as follows: there are one (for one cell type study) or multiple main micro-channels (for multicellular research). Each of the main channels comprises two parallel rows of regularly distributed micro-sized posts (shown in Figure 1) to separate one channel from another. The purpose of using micro-posts is to preserve some space for the cell growth medium to enter and also give rise to the observation of cell–cell communication and interaction. Even though these designs worked very well for cell studies with microfluidic chips made of PDMS, it is difficult to scale up the production of the chips and hence the commercialization. One of the reasons is that PDMS is expensive, and it is mostly impossible to scale up the massive production [10,11]. Another reason is that the geometries of these sharp-edge micro-posts in the previous works (trapezoid [4,8,12], hexagon [5]) can give rise to difficulty when using micromachining fabrication methods such as micro-milling [11]. Moreover, to the authors’ knowledge, the shape and spacing distance of the micro-posts together have not yet been optimized for the current reported work on the PAH-on-a-chip [4] and most of the other published works [5,6,12,13,14].

In this paper, we perform both experimental and theoretical studies on the design and fabrication of microfluidic chips for cell seedings and multicellular cell culture applications. Particularly, we redesign, fabricate, and conduct experiments to evaluate the performance of multi-channel microfluidic chips in terms of preventing liquid migration from one channel to another with three different micro-post shapes: trapezoid, hexagon, and circle. We conduct cell seeding and culturing experiments in the circular micro-post chips for the first time. Our experimental results and the COMSOL Multiphysics simulation showed for the first time that the circular micro-posts channel performed excellently to prevent liquid and cell leakage from one channel to another. The distance between the micro-posts is also optimized in the design, fabrication, and analytical studies. We also conduct computer-aided manufacturing (CAM) simulation to confirm that the fabrication of circular-shape micro-posts in a microfluidic master mold is much easier and more rapid than other multiple-edging structures such as the hexagonal posts when utilizing micro-machining techniques, e.g., micro-milling.

## 2. Materials and Methods

### 2.1. Chip Fabrication

In order to investigate and compare the performance of different shapes of the micro-posts, i.e., trapezoidal, hexagonal, and circular ones from this work to the previous in ref [4], as well as to other previous studies where the chips were made of PDMS [5,8], we fabricated the microfluidic chips from PDMS soft-photolithography. AutoCAD 2020 software was used to design the layout of the microfluidic channels with the three aforementioned shapes for the micro-posts. The distances between the micro-posts also varied from 200 μm to 100 μm to evaluate the performance of the chip in terms of preventing fluid leakage from one channel to another. The designed layout was sent to (CAD/Art Services, Inc., Bandon, OR, USA) for printing out the plastic photomasks, which were used later in the UV exposure step with the UV-KUB 2, Kloe, France. Photoresist SU8 2100 (from Kayaku Advanced Material Inc., Westborough, MA, USA) was spin-coated on a 4-inch silicon wafer (from University Wafer Inc., South Boston, MA, USA) using the spin coater WS 650HZB (Laurell Technologies Corporation, North Wales, PA, USA) to achieve a thickness of 150 μm. Hard and soft bake steps were done with the Teca AHP solid-state heat/cool machine from Thermoelectric Cooling America Corporation, Chicago, IL, USA. The master mold was then coated with an anti-sticking layer via a silanization step under vacuum for 1.5 h using chlorotriethylsilane (CTMS) 98% (Sigma, Ronkonkoma, NY, USA). DOW SYLGARD™ 184 silicone was used for the PDMS casting with a 10:1 mix ratio. The PDMS part is then bonded with a coverslip to seal the channels using ambient plasma treatment for 2 min (PDC-001-HP series, Harrick plasma, Ithaca, NY, USA) at medium RF power. The biochips were then stored in an oven at 80 °C for 2 h to enhance the bonding. Subsequently, the chips were treated with UV (UV Light Box Benchtop Decontamination Chambers, Air Science Inc., Fort Myers, FL, USA) for 45 min to sterilize before proceeding to the cell seeding steps.

### 2.2. Cell Seedings

Prior to the cell seeding, the microchannels were coated with Poly-D-Lysine (PDL) solution (2 mg/mL) to enhance the gel adhesion to the channel. After infusing PDL into the channels, the chips were kept in the incubator for 4 h and then PDL was removed by thoroughly washing the channels with sterilized water. The chips were kept in the oven for 48 h at 80 °C and then cell seeding was performed. 

**Figure 2:**
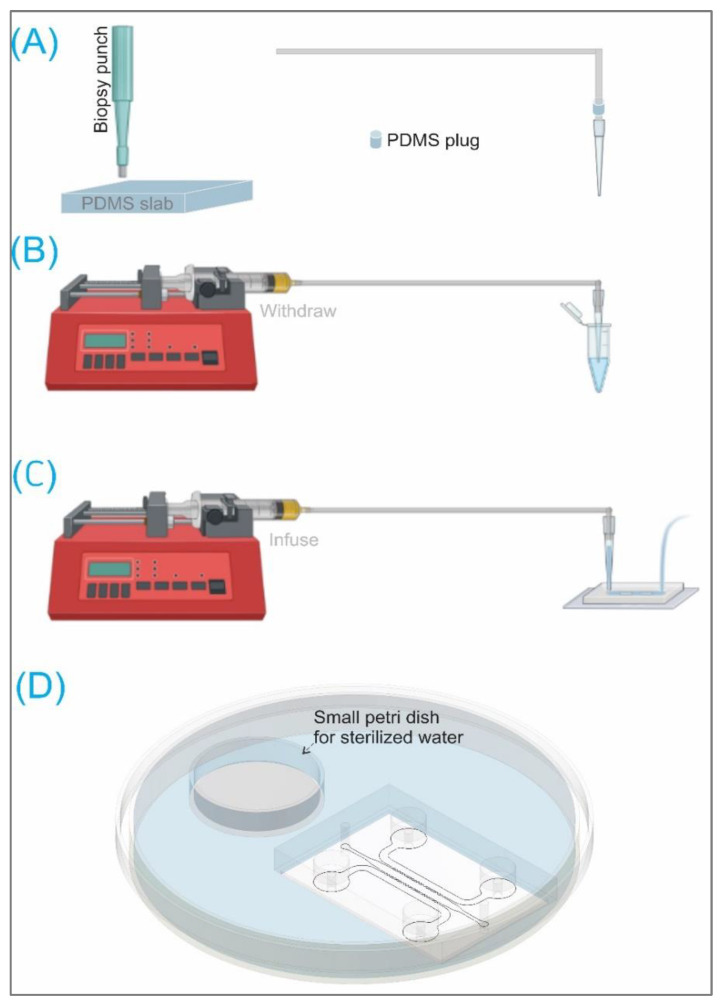
Schematics of: (**A**) Fabricating a PDMS plug used to connect a Cole-Parmer PTFE microbore tubing 0.012″ ID and a pipette tip. (**B**) Withdrawing cell suspension using a (New Era Pump) syringe pump and tubings. (**C**) Infusing cell suspension. (**D**) A tissue chip in a large petri dish containing a smaller petri dish having sterilized water acting as a humidifier for cell seeding in an incubator at 37 °C with 5% CO_2_. Figure partially made using Biorender.com.

In order to investigate the ability of using circular micro-posts, smooth muscle cell with a density of 10^6^ cell/mL was loaded into the central channel with and without collagen gel. The cell suspension, with or without gel, was withdrawn from a vial using a syringe pump (New Era Pump Systems Inc., Farmingdale, NY, USA) at the flow rate of 15 μL/min and 50 μL/min, respectively (Figure 2B). These flow rates are comparable to other previous works [15,16]. The cell suspension (with or without gel) was then infused into the microchannel with the same flow rate as the withdrawing (Figure 2c). A PDMS plug made from punching a PDMS slab with a 6 mm puncher was used to interface the tubing (Cole-Parmer PTFE microbore tubing, 0.012″ ID × 0.030″ OD) and the pipette tip (Figure 2A–C). We developed this method using a pipette tip connecting to a syringe pump to efficiently save cells and medium since those materials are not in the syringe or the tubings, but only in the pipette tip. After seeding, the chips were placed in a large petri dish, acting as a humidifier, with a small petri dish containing water. The chip’s humidifier was kept in the incubator at 37 °C with 5% CO_2_ (Figure 2D).

### 2.3. COMSOL Multiphysics Simulation

COMSOL Multiphysics 5.6 was used to simulate the fluid flow inside the microchannel to compare the performance of circular posts to the hexagonal posts in terms of preventing leakage. The simulation was conducted on 2D images extracted from the chip layout designed in AutoCAD. For the purpose of simulating the flow pattern inside the channel, without losing generality, we considered the microfluidic chip made of 3 channels, in which the middle channel comprises two parallel rows of micro-posts. From the experimental flow rate of 15 μL/min, the velocity at the inlet is set at 0.0016 m/s.

### 2.4. Analytical Solutions

In our biochip designs inspired by other works [5,8], the microfluidic channel possesses a system of regularly spaced posts that act as geometric capillary burst valves enabling the interface between cell–media and cell–cell. The pressure variations at the liquid interface are crucial in deciding whether leakage will occur, and maintaining the integrity of the liquid interface between neighboring channels requires a balance of surface tension and capillary pressures throughout the liquid injection process. Leakage will not occur if the pressure differential ΔP (=ΔP_d_ − ΔP_w_) reaches a minimum threshold, in which ΔP_d_ and ΔP_w_ are the pressure difference between the gap spacing (d) and channel width (w), respectively [5]. In the previous work in ref [4], we had not yet optimized the spacing distance among the posts. In this work, Maple 2021 was used to plot the dependence of the spacing distance on the ΔP. We also performed further mathematical analysis to assess the feasibility of using circular micro-posts.

### 2.5. CAM Simulation

3D design of the chips from Autodesk Inventor 2020 was imported into Cimatron 14 for CAM simulation to understand the milling process of the circular and hexagonal micro-posts. The diameter of the circular post was 300 μm, which is equal to the one for the hexagon. The end mill with 300 μm was chosen to match the diameters of the circular and the hexagonal posts.

## 3. Results and Discussions

### 3.1. Analytical Solution

**Figure 3:**
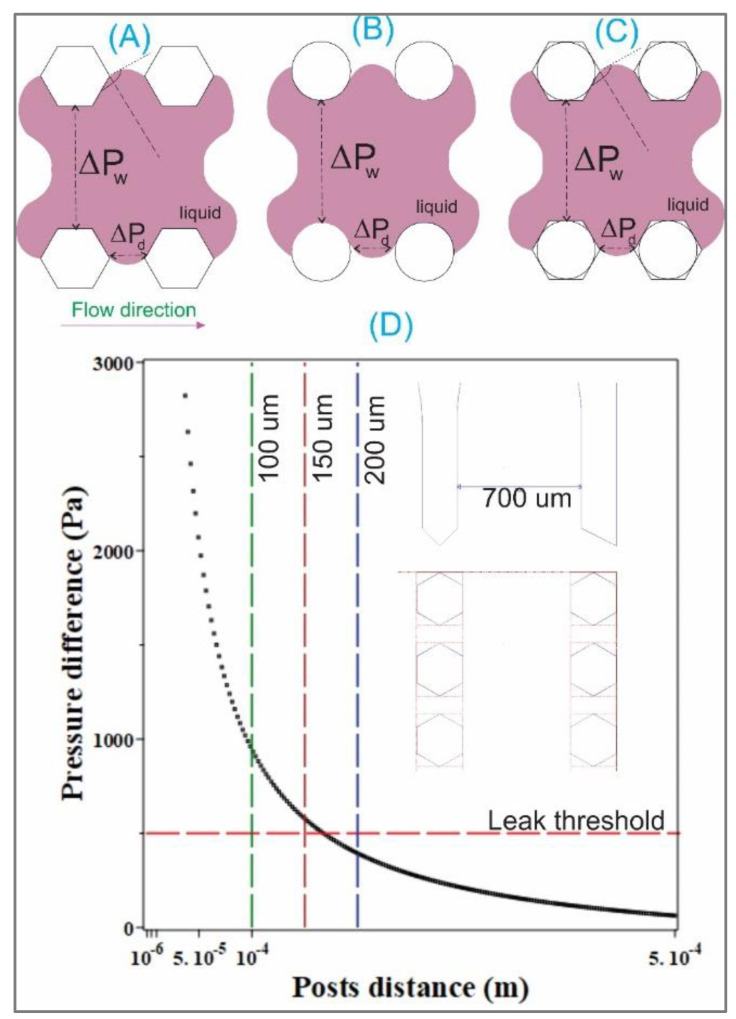
Schematics to illustrate the pressure differential ΔP_d_ and ΔP_w_ for cases of using (**A**) hexagonal and (**B**) circular micro-posts. (**A**) is inspired by the work of Huang, C.P., et al. [5], (**B**) for the circular post is our new design. (**C**) Illustration for the imposition of a circle inscribed on a hexagonal post to estimate the contact angle with the wall of the infused liquid. (**D**) ΔP = ΔP_d_ − ΔP_w_ as a function of spacing distance, inspired and modified by Huang, C.P., et al. [5], applied for chips with channels having 700 μm width. Figure 4, on the next page, denotes and illustrates our new theory.

Figure 3A,B illustrate the pressure differential ΔP = ΔP_d_ − ΔP_w_ for cases using hexagonal and circular micro-posts, respectively. 

It can be seen from Figure 3D that for the microchannels with the width w = 700 μ, the spacing distance at 200 μm can result in leakage as the ΔP is below the threshold pressure (setting at 500 Pa for water, i.e., dynamic viscosity η = 10^−3^ Pa·s, density ρ = 10^3^ Kg/m^3^ [5]).

**Figure 4:**
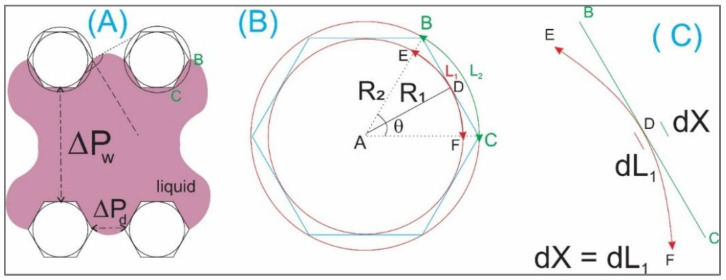
(**A**,**B**) denote parameters for analysis of the similarity of a hexagon and its inscribed and circumscribed circles. (**C**) An enlarged image of the curves at the water contact line.

Figure 3C illustrates an imposition of a circle inscribed on a hexagonal post to estimate the contact angle with the wall of the infused liquid. Figure 4A,B denotes parameters for analysis of the similarity of a hexagon and its inscribed and circumscribed circles. It can be seen from Figure 4B that:

(1) $$\begin{array}{l} R_{2}=\frac{L_{2}}{\theta }\\ R_{1}=\frac{L_{1}}{\theta } \end{array}$$

The length of the line BC (water contact line Figure 4A,B) has its value of x, we have the following.

(2) $$x=2\sqrt{(R_{2}^{2}-R_{1}^{2}})$$

From Equations (1) and (2), we obtain:

(3) $$x=2\sqrt{\frac{L_{2}^{2}}{\theta^{2}}-\frac{L_{1}^{2}}{\theta^{2}}}=\frac{2}{\theta }\sqrt{\left(L_{2}+L_{1})(L_{2}-L_{1}\right)}$$

The curve $\overset{⌢}{EF}$ crosses the line BC at the center point D. From D, considering a small fraction distance dx and dL_1_ (Figure 4C), we have

(4) $$\begin{array}{l} dx\approx dL_{1}\\ dL_{1}+dL_{2}\approx 2dL_{1} \end{array}$$

For small structures (in microscale), i.e., microstructures, Equation (4) can be rewritten as

(5) $$\begin{array}{l} x\approx L_{1}\\ L_{1}+L_{2}\approx 2L_{1} \end{array}$$

We have Equation (3) as follows

(6) $$L_{1}=\frac{2\sqrt{2}}{\theta }\sqrt{L_{1}(L_{2}-L_{1})}$$

Or:

(7) $$\begin{array}{l} L_{1}=\frac{8}{\theta^{2}}(L_{2}-L_{1})\\ \to \frac{L_{1}(1+\frac{8}{\theta^{2}})}{\left(\frac{8}{\theta^{2}}\right)}=L_{2} \end{array}$$

Since $x\approx L_{1}$ (Equation (5)), *θ* is hence small enough, we have

$$\frac{8}{\theta^{2}}\gg 1;\to 1+\frac{8}{\theta^{2}}\approx \frac{8}{\theta^{2}}$$

(8) $$\to \frac{1+\frac{8}{\theta^{2}}}{\frac{8}{\theta^{2}}}\approx 1$$

Equation (7) is simplified to

(9) $$L_{2}=L_{1}$$

Or from Equations (5) and (9), we obtain:

(10) $$x=L_{2}=L_{1}$$

In other words, the water contact lines, hence the water contact surfaces for three cases, are appropriately equal. In other words, this analysis confirms that the utilization of circular micro-posts is feasible in the tissue chips.

### 3.2. Chip Fabrications

For the first time, we fabricated various types of five-channel tissue chips with three different micro-post shapes: trapezoid, hexagon, and circle. In these five channels, each of the three channels in the middle of the chips comprises two parallel rows of micro-posts for cell seeding, and the rest two outmost channels are for filling with cell media. Figure 1 shows the typical design for trapezoid micro-post five-channel chips. Hexagonal and circular micro-post chips share similarities in the layouts of the chip in Figure 1, except the shapes of the micro-posts are changed from trapezoid to hexagon or circle. The spacing distance between the micro-posts also varies from 200 μm to 100 µm to optimize and eliminate liquid leakage.

**Figure 5:**
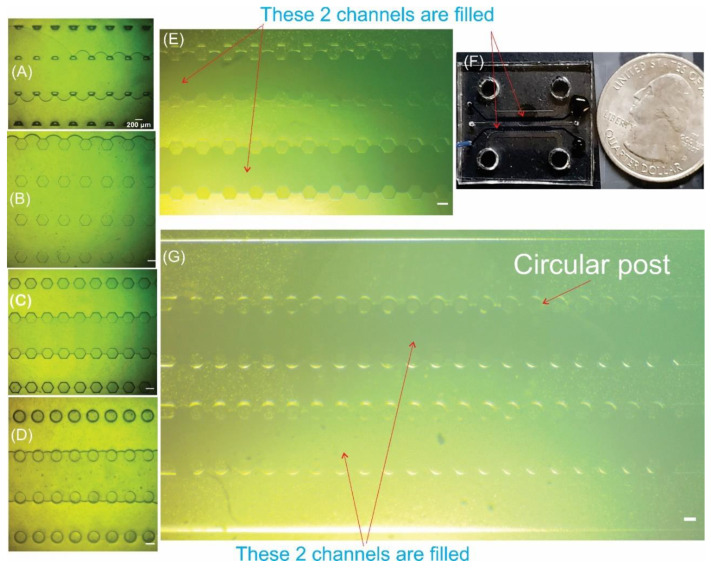
Microscope images of leakage from the middle channel to the neighboring compartments during liquid infusion using chips with 200 µm spacing distance for (**A**) trapezoidal posts and (**B**) hexagonal posts. Successful infusion of middle channels when using chips with 100 µm spacing distance for (**C**) hexagonal posts and (**D**) circular posts. Successful infusing multiple side channels for chips with 100 µm spacing distance for (**E**,**F**) hexagonal posts and (**G**) circular posts.

Figure 5F shows a digital image of a five-channel tissue chip with hexagonal posts, and channels 2 and 4 are filled with trypan blue for testing liquid leakage (also shown in the microscope image in Figure 5E). The chip is successfully filled without leakage since the spacing distance of the micro-posts is 100 µm. As can be seen in Figure 5G, the circular micro-posts offer the same barrier effect to prevent leakage as the hexagonal posts. A larger spacing distance (200 µm) resulted in leakage (shown in Figure 5A,B for hexagonal and trapezoidal posts). The experimental results are hence consistent with the analytical prediction shown in the previous Section 3.1. A video clip that records the filling process for the microchannel in Figure 5G is available in the Supplementary Materials, Media S1. Figure 5C,D shows the successful filling of the middle channel (channel number 3) with 2 mg/mL gel (type 1 collagen). 

Furthermore, SMC cell seeding in the gel was performed in the experiment shown in Figure 5D. The SMC cell image after six days of incubating is shown in Figure S1 in the Supplementary Materials.

### 3.3. COMSOL Simulations

**Figure 6:**
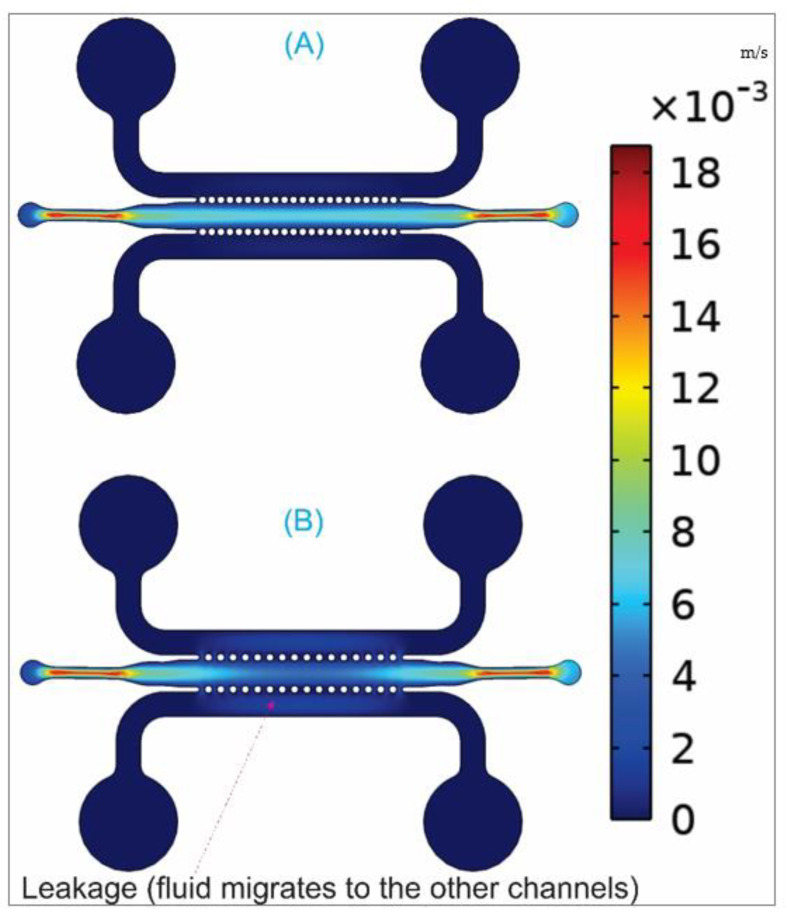
COMSOL simulation results showing the effect of spacing distance in controlling fluid leakage. Leakage does not occur at a small spacing distance (**A**) 70 μm, but at a large spacing distance (**B**), 200 µm.

Figure 6 shows the velocity (m/s) patterns of the fluid flowing inside the microchannels made of circular micro-posts to simulate the leakage. The color legend presents the variety of fluid velocities starting from 0 m/s (blue color) and increasing to a maximum with red color. 

The results indicate that for the spacing distance smaller than 100 µm (Figure 6A), the leakage does not occur in comparison with the spacing distance of 200 μm (Figure 6B). 

These simulation results are consistent with the analytical solution and experimental observation shown in the previous sections (Figure 3 and Figure 5). Furthermore, it is well-known that circular and smooth curvature is good for cell seeding (and cell attachment) compared to sharp-edge structures [13,17].

### 3.4. CAM Simulations

**Figure 7:**
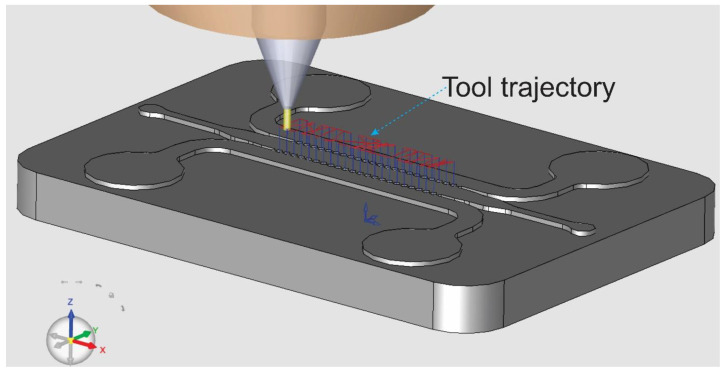
Captured image from Cimatron 14 on the simulation process of fabricating a master mold for injection molding of circular micro-post tissue chip. For the simulation video, see Supplementary Materials, Media S2.

As shown in the previous sections with experiments, simulations, and in-depth analysis, circular micro-posts can be used in place of other shapes (trapezoid, hexagon). It is important to emphasize that using circular micro-posts opens up the possibility for rapid and cost-efficient fabrication of tissue chips. This paper demonstrates that for the first time. This is because, for rapid micro-machining fabrication methods such as micro-milling and injection molding, round shapes are much easier and more rapid to fabricate compared to sharp-edged micro-structures [11]. The CAM simulation (illustrated in Figure 7) in this section is conducted using Cimatron 14 for the milling tip of 0.3 mm diameter to mill the arrays of circular micro-posts in the tissue chip master mold. In the Supplementary Materials, Media S2 presents the milling process. It shows that the milling process for fabricating the round micro-posts is robust, rapid, and easy. It is impossible to mill the hexagonal structures using the same end mill (with a diameter of 0.3 mm). The same milling methods can also be used to fabricate the tissue chip directly from PMMA plates instead of making a master mold for polymer injection molding. Both fabrication methods are rapid and cost-efficient compared to the expensive and time-consuming clean-room photolithography (Table S1). 

## 4. Conclusions

We demonstrated, for the first time, both experimentally (by designing, fabricating, testing, and using) and theoretically (by providing analytical solutions and simulations (COMSOL, CAM)) that circular micro-posts can be used in place of other shapes (such as hexagon, trapezoid) in tissue chips for multicellular cell cultures. The utilization of circular micro-posts in the tissue chips not only offers more accessible environments for cells to attach, seed, and grow compared to other sharp-edged structures but also opens up the possibility for a rapid, cost-efficient, and massive production of the chips via micromachining techniques such as micro-milling, which is the technique used to produce a master mold for polymer injection molding, hence large-scale fabrication. 

## Data Availability

Not applicable.

## Supplementary Materials

The following supporting information can be downloaded at: https://www.mdpi.com/article/10.3390/bios12080587/s1, Figure S1: SMC cell after 6 days of seeding in the circular micro-post tissue chips. Scale bar 200 µm; Table S1: Advantages of using circular posts; Media S1: Filling circular micro-post tissue chips; Media S2: CAM simulation of milling process for fabricating circular micro-post in the master mold.

## References

[1] Myers D.R., Sakurai Y., Tran R., Ahn B., Hardy E.T., Mannino R., Kita A., Tsai M., Lam W.A. (2012). Endothelialized microfluidics for studying microvascular interactions in hematologic diseases. J. Vis. Exp..

[2] Xu S., Li X., Liu Y., He P. (2016). Development and Characterization of In Vitro Microvessel Network and Quantitative Measurements of Endothelial [Ca2+]i and Nitric Oxide Production. J. Vis. Exp..

[3] Mannino R.G., Qiu Y., Lam W.A. (2018). Endothelial cell culture in microfluidic devices for investigating microvascular processes. Biomicrofluidics.

[4] Al-Hilal T.A., Keshavarz A., Kadry H., Lahooti B., Al-Obaida A., Ding Z., Li W., Kamm R., McMurtry I.F., Lahm T. (2020). Pulmonary-arterial-hypertension (PAH)-on-a-chip: Fabrication, validation and application. Lab Chip.

[5] Huang C.P., Lu J., Seon H., Lee A.P., Flanagan L.A., Kim H.Y., Putnam A.J., Jeon N.L. (2009). Engineering microscale cellular niches for three-dimensional multicellular co-cultures. Lab Chip.

[6] Kim S., Lee H., Chung M., Jeon N.L. (2013). Engineering of functional, perfusable 3D microvascular networks on a chip. Lab Chip.

[7] Ko J., Lee Y., Lee S., Lee S.R., Jeon N.L. (2019). Human Ocular Angiogenesis-Inspired Vascular Models on an Injection-Molded Microfluidic Chip. Adv. Healthc. Mater..

[8] Shin Y., Han S., Jeon J.S., Yamamoto K., Zervantonakis I.K., Sudo R., Kamm R.D., Chung S. (2012). Microfluidic assay for simultaneous culture of multiple cell types on surfaces or within hydrogels. Nat. Protoc..

[9] Chung S., Sudo R., Mack P.J., Wan C.-R., Vickerman V., Kamm R.D. (2009). Cell migration into scaffolds under co-culture conditions in a microfluidic platform. Lab Chip.

[10] Nguyen T., Chidambara V.A., Andreasen S.Z., Golabi M., Huynh V.N., Linh Q.T., Bang D.D., Wolff A. (2020). Point-of-care devices for pathogen detections: The three most important factors to realise towards commercialization. TrAC Trends Anal. Chem..

[11] Nguyen T., Chidambara Vinayaka A., Duong Bang D., Wolff A. (2019). A Complete Protocol for Rapid and Low-Cost Fabrication of Polymer Microfluidic Chips Containing Three-Dimensional Microstructures Used in Point-of-Care Devices. Micromachines.

[12] Hajal C., Offeddu G.S., Shin Y., Zhang S., Morozova O., Hickman D., Knutson C.G., Kamm R.D. (2022). Engineered human blood-brain barrier microfluidic model for vascular permeability analyses. Nat. Protoc..

[13] Hyung S., Lee S.R., Kim J., Kim Y., Kim S., Kim H.N., Jeon N.L. (2021). A 3D disease and regeneration model of peripheral nervous system-on-a-chip. Sci. Adv..

[14] Farahat W.A., Wood L.B., Zervantonakis I.K., Schor A., Ong S., Neal D., Kamm R.D., Asada H.H. (2012). Ensemble analysis of angiogenic growth in three-dimensional microfluidic cell cultures. PLoS ONE.

[15] Ezra Tsur E., Zimerman M., Maor I., Elrich A., Nahmias Y. (2017). Microfluidic Concentric Gradient Generator Design for High-Throughput Cell-Based Studies. Front. Bioeng. Biotechnol..

[16] Manbachi A., Shrivastava S., Cioffi M., Chung B.G., Moretti M., Demirci U., Yliperttula M., Khademhosseini A. (2008). Microcirculation within grooved substrates regulates cell positioning and cell docking inside microfluidic channels. Lab Chip.

[17] Cho M., Park J.K. (2021). Modular 3D In Vitro Artery-Mimicking Multichannel System for Recapitulating Vascular Stenosis and Inflammation. Micromachines.

