# Supplementary material for: Multicellular Cell Seeding on a Chip: New Design and Optimization towards Commercialization
Source: Biosensors (Basel). 2022 Aug 1;12(8):587. doi: 10.3390/bios12080587 (PMC9405756; doi:10.3390/bios12080587)
Supplement: Supplementary file 1 [file biosensors-12-00587-s001.zip › biosensors-1806671-supplementary.pdf]

Supplementary Materials

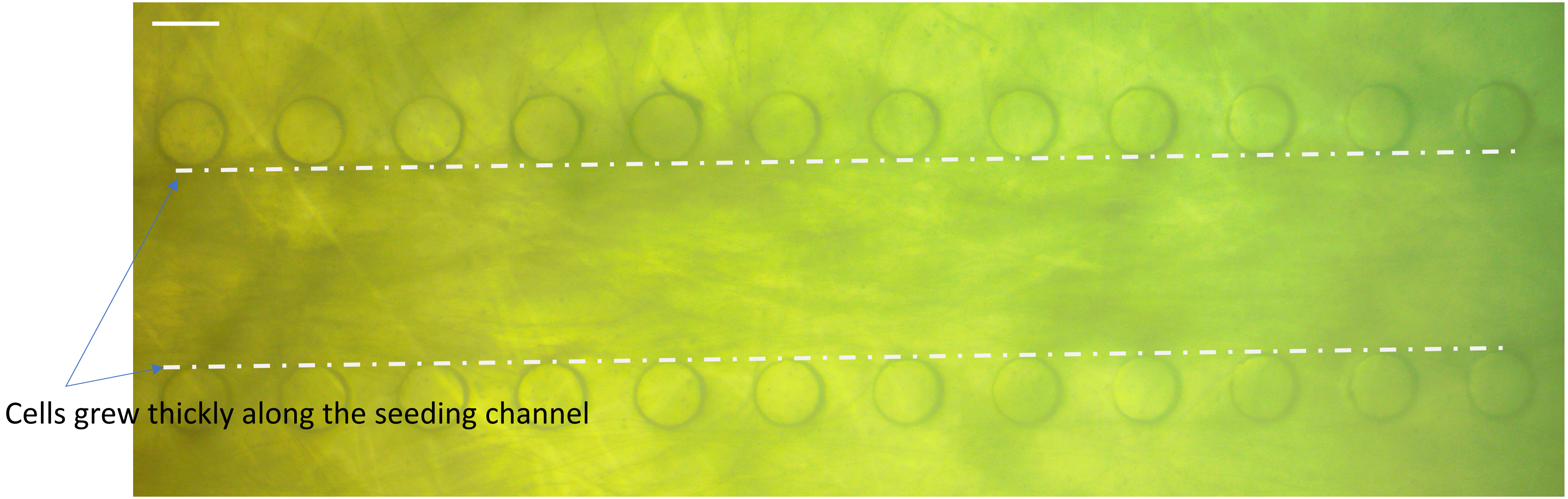

Figure S1: SMC cell after 6 days of seeding in the circular micro-post tissue chips. Scale bar 200 μm

Table S1: Advantages of using circular posts

Design	Reference	Possible fabrications	Scaling up production	Cost-efficient
**Circular posts**	First time reported in our work (this manuscript)	Micro-milling and polymer injection moulding	Yes	Yes
**Hexagonal posts**	[5]	Soft photolithography	No	No
**Trapezoidal posts**	[4, 8, 12]	Soft photolithography	No	No

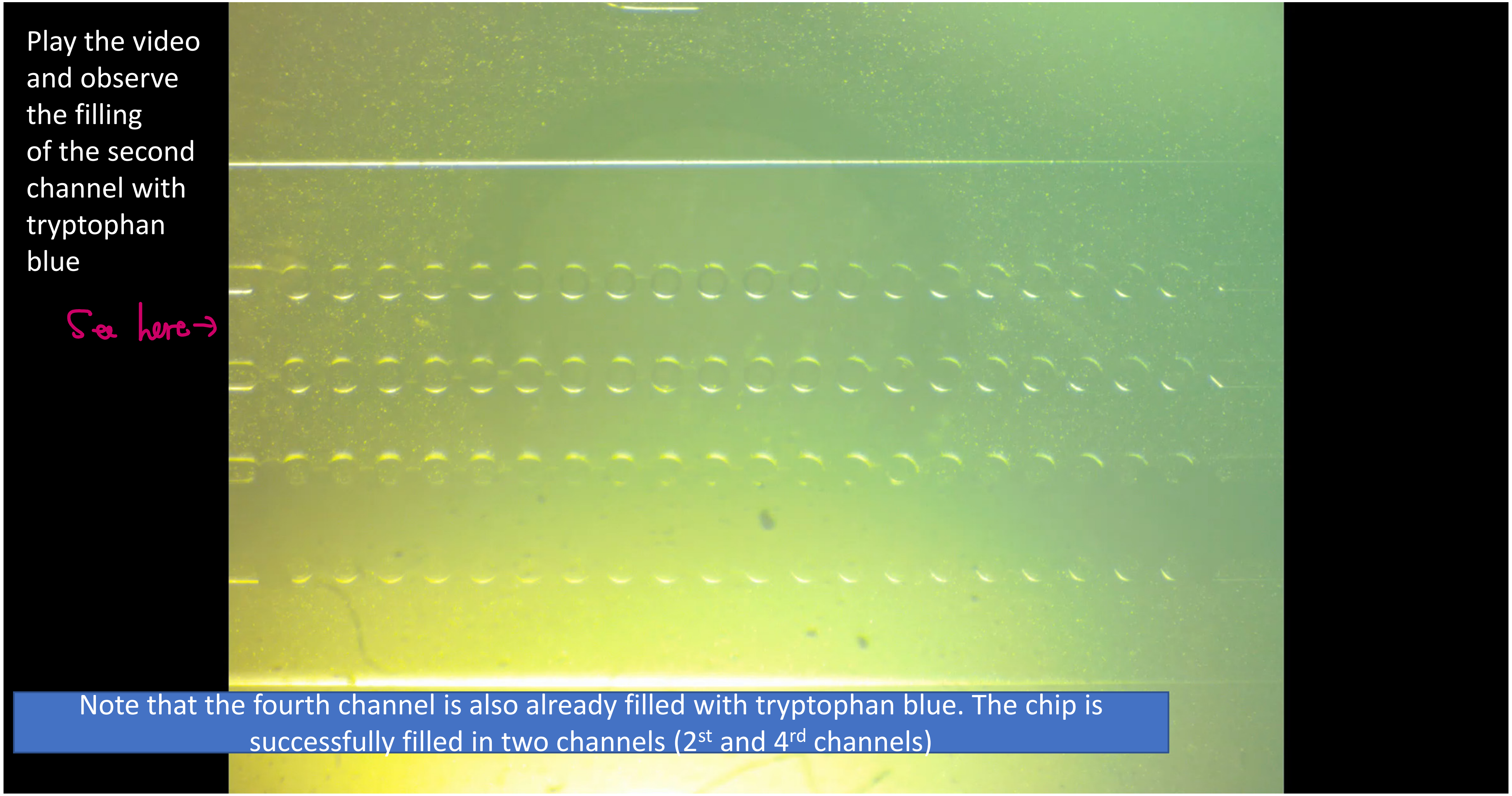

Media S1: Filling circular micro-post tissue chips

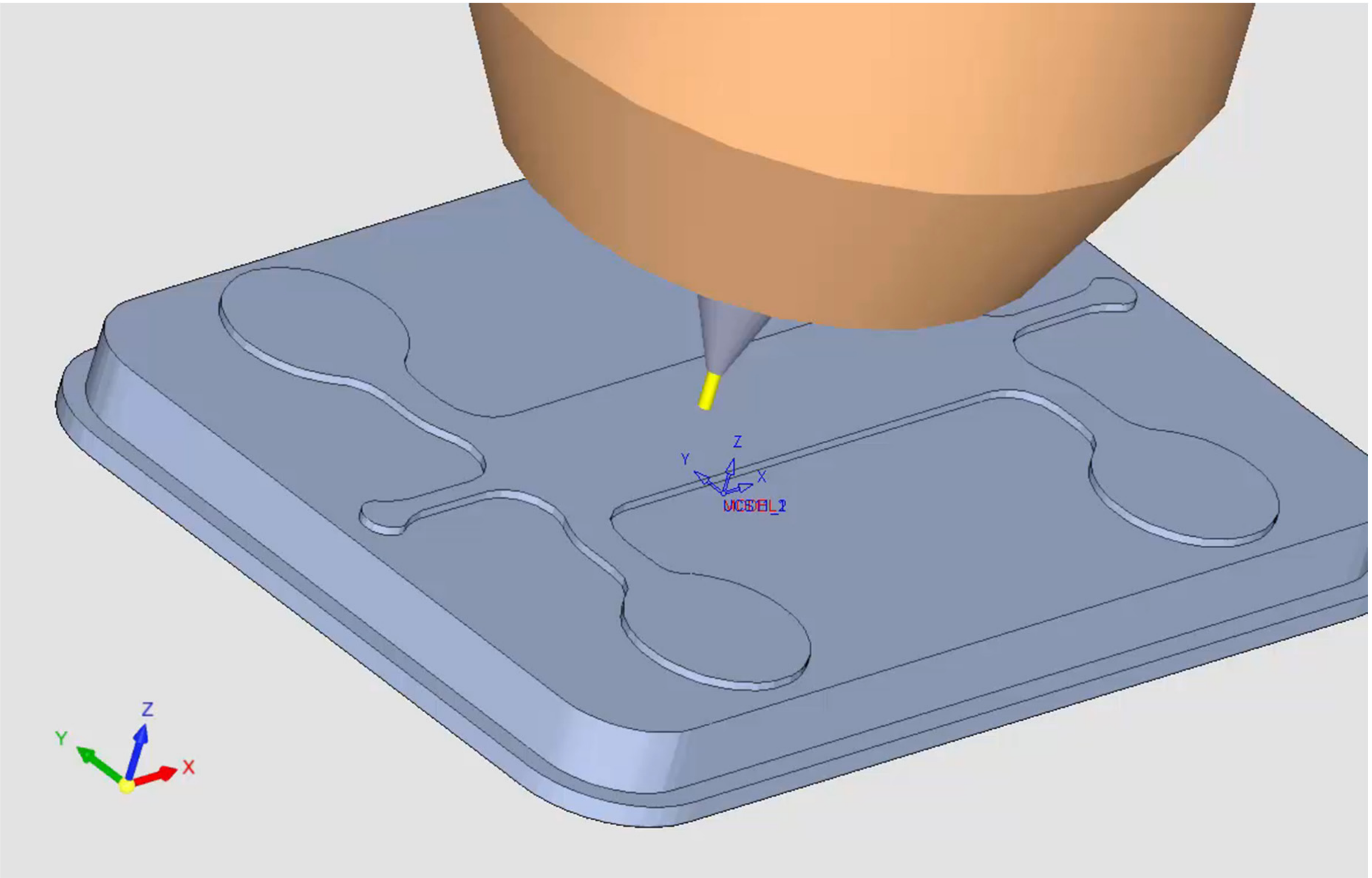

Media S2: CAM simulation of milling process for fabricating circular micro-post in the master mold
